# Endometriosis Coinciding with Uterus Didelphys and Renal Agenesis: A Literature Review

**DOI:** 10.3390/jcm13247530

**Published:** 2024-12-11

**Authors:** Davut Dayan, Florian Ebner, Wolfgang Janni, Katharina Hancke, Duygu Adiyaman, Beate Huener, Michelle Hensel, Andreas Daniel Hartkopf, Marinus Schmid, Stefan Lukac

**Affiliations:** 1 Klinik für Frauenheilkunde und Geburtshilfe, Universitätsklinikum Ulm, 89075 Ulm, Germany; dr.ebner@web.de (F.E.); wolfgang.janni@uniklinik-ulm.de (W.J.); katharina.hancke@uniklinik-ulm.de (K.H.); duygu.adiyaman@uniklinik-ulm.de (D.A.); beate.huener@uniklinik-ulm.de (B.H.); michelle.hensel@uniklinik-ulm.de (M.H.); andreas.hartkopf@med.uni-tuebingen.de (A.D.H.); marinus.schmid@uniklinik-ulm.de (M.S.);; 2 Abteilung für Frauenheilkunde und Geburtshilfe, Alb-Donau Klinikum Ehingen, 89584 Ehingen (Donau), Germany; 3 Department für Frauengesundheit, Universitäts-Frauenklinik Tübingen, 72076 Tübingen, Germany

**Keywords:** endometriosis, uterus didelphys, genitourinary malformation, Herlyn–Werner–Wunderlich syndrome, OHVIRA syndrome

## Abstract

**Background/Objectives**: Endometriosis and urogenital malformation with uterus didelphys and renal agenesis might occur concomitantly, and the question arises whether both entities are associated with each other. **Methods**: A literature search was conducted in PubMed and Web of Science, using the following search terms: “endometriosis and uterine malformation, endometriosis and Herlyn–Werner–Wunderlich syndrome”, “endometriosis and OHVIRA (Obstructed Hemivagina and Ipsilateral Renal Anomaly) syndrome” and “uterus didelphys, renal agenesis and endometriosis”. **Results**: We identified and examined 36 studies, comprising a total of 563 cases with coinciding endometriosis and OHVIRA. The most prevalent symptoms were dysmenorrhea and lower abdominal pain. Renal agenesis occurred more frequently on the right side. In the majority of cases, vaginal septum resection was performed to alleviate hematometrocolpos. Among the 97 cases necessitating abdominal exploration, endometriosis was identified in 61 patients (62.9%), although this figure is most likely an overestimation. However, a significantly heightened risk of endometriosis was evident. **Conclusions**: This literature review highlights the importance of considering the potential for urogenital malformation and endometriosis in cases of dysmenorrhea during adolescence. Ultrasound examination has proven to be a valuable diagnostic tool for identifying uterine abnormalities and guiding subsequent diagnostic and, if necessary, surgical interventions. Thorough assessment and appropriate management are imperative to mitigating the long-term consequences associated with deep infiltrating endometriosis.

## 1. Background

Endometriosis is a benign, chronic condition characterized by the development of endometrial-like lesions outside the uterine cavity. Endometriosis occurs in approximately 10% of women of reproductive age and can significantly impair quality of life and fertility [1,2,3]. Endometriosis risk factors include genetic, immunological and environmental factors [4], but the etiology has not yet been conclusively determined. A widely accepted cause is Sampson’s retrograde menstruation theory, proposed more than 100 years ago [5]. This phenomenon is more prevalent in the presence of obstructive uterine anomalies. Several studies have established a correlation between such anomalies and the development of endometriosis [6,7,8,9,10]. While retrograde menstruation could account for the pathogenesis of endometriosis, additional evidence suggests that immunological or genetic factors might also contribute [11,12]. To date, the prevalence of endometriosis in adolescents remains uncertain. In a retrospective study, conducted by Haas et al., involving 42,079 histologically confirmed endometriosis cases, 0.05% were patients aged 10 to 15 years and 1.93% patients aged 15 to 20 years. Notably, the proportion of patients increased in higher age groups, with 6.11% of cases referring to patients aged 20 to 25 years and 78.37% to patients aged 20 to 45 years [13]. In line with this study, Eisenberg et al. observed a 0.8% endometriosis rate in adolescents between 15 and 19 years in their retrospective analysis of 2 million patients [14].

A rare Müllerian duct anomaly is the congenital Obstructed Hemivagina and Ipsilateral Renal Anomaly, first described in 1922. This condition is known as Herlyn–Werner–Wunderlich syndrome (HWWS), named after the authors who reported the initial cases between 1971 and 1976, or as spacing OHVIRA syndrome—an acronym that summarizes the main features of the condition. These patients show uterus didelphys, ipsilateral vaginal obstruction and ipsilateral renal agenesis [15,16,17]. The syndrome arises from aberrant development of the paramesonephric (Müllerian) and mesonephric (Wolffian) ducts [15]. Anomalies stem from disruptions in the formation, canalization, fusion or absorption of the Müllerian ducts during the 8th to 12th week of embryonic development [18,19,20]. Although the precise prevalence remains uncertain, current estimates range from 0.1% to 3.8% [16]. The severity of these anomalies varies and depends on the extent of the development of the Müllerian system. The standardized terminology of the American Society for Reproductive Medicine (ASRM) classification is used to precisely identify each anomaly and to simplify the communication of these anomalies [21]. One of the most important symptoms is cyclical lower abdominal pain after menarche and, in the case of vaginal obstruction, pelvic distension [17,20,22,23].

In a recent literature review on HWWS, Liu et al. assessed 1673 HWWS patients presenting with symptoms and anomalies [24]. The most common symptoms were dysmenorrhea (53.8%), abnormal uterine bleeding (28.9%) and vaginal discharge (26.6%). Frequently observed anomalies included a right-obstructed hemivagina (57.3%), hematocolpos (81.7%), uterus didelphys (88.8%) and ipsilateral renal agenesis (93.1%). Vaginal septum excision was the primary treatment (91.8%), with minimally invasive surgery performed in 48.5% of cases, often following vaginal surgery (61.9%). Endometriosis was identified in 9.6% of these patients, with 52% involving ipsilateral ovarian endometriotic cysts.

Endometriosis is not universally more common in patients with Müllerian anomalies, but it is more frequently seen in those with outflow obstructions, such as hematosalpinx, hematometra or hematocolpos. An association between OHVIRA and deep infiltrating endometriosis (DIE) has been reported [20,23,25]. In a recent case report by Choridah and Pangastuti, an adolescent patient with cyclic pain and an endometriotic cyst on one side, accompanied by hemivaginal obstruction and several endometriotic nodules on the peritoneum and diaphragm, presented and didelphys was diagnosed by MRI [26]. In another recent case series, Takahashi et al. analyzed 12 adolescent patients who had primary surgery for obstructive Müllerian anomalies and 31 patients in the same age group who underwent surgery for ovarian endometrioma. Of the patients with obstructive Müllerian anomalies, including four cases of Herlyn–Werner–Wunderlich syndrome, two cases with a non-communicating functional uterine horn and two cases of cervical aplasia, 50% developed endometriosis [27].

Some studies have correlated the presence of Müllerian duct malformations with the incidence of severe endometriosis [23,28]. An increased risk of endometriosis in uterine malformation has not yet been conclusively determined; however, it is theorized that obstruction of the genital tract results in increased retrograde menstruation, thereby leading to the development of endometriosis [8]. Another possible explanation is that a family history of these two conditions predisposes individuals who suffer from one to the occurrence of the other [29]. Although the link between pelvic endometriosis and HWWS is still not well understood, Sampson’s theory of retrograde menstruation and implantation is commonly accepted but does not account for all cases of pelvic endometriosis. Detecting an association between endometriosis and OHVIRA, alongside identifying overlapping symptoms, might help with the early identification of patients at particular risk of impaired fertility and diminished quality of life and the selection of corresponding, therapeutic options.

Here we review the literature on previous reports of both entities coinciding in adolescent patients. The available data highlight the need for the timely diagnosis of uterine malformations possibly associated with endometriosis in adolescent patients with cyclical abdominal pain.

## 2. Methods

A systematic literature review was conducted to assess the available literature on endometriosis co-occurring with uterine malformations in general and OHVIRA in particular. The PubMed and Web of Science databases were searched for relevant publications, using the following search terms: “endometriosis AND uterine malformation”, “endometriosis AND Herlyn–Werner–Wunderlich syndrome”, “endometriosis AND OHVIRA (Obstructed Hemivagina and Ipsilateral Renal Anomaly) syndrome” and “uterus didelphys, renal agenesis AND endometriosis”. The search was conducted without limits and adhered to the PRISMA (Preferred Reporting Items for Systematic Reviews and Meta-Analyses) guidelines [30]. This systematic review was registered in the Prospero database, under the registration number CRD42023462124. Two independent reviewers evaluated the suitability of papers for inclusion in the systematic review. Exclusion criteria encompassed papers not written in English, video material, conference abstracts, editorial letters, reviews and studies lacking complete or original data.

**Figure 1:**
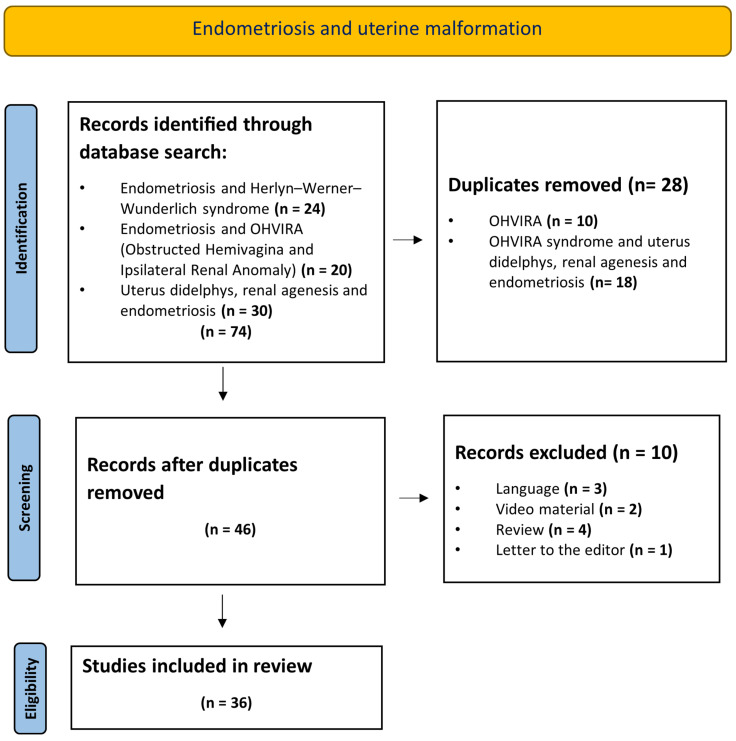
Flowchart of study identification, screening, eligibility and inclusion.

A total of 664 studies were identified using the search term “endometriosis and uterine malformation”. However, many of these studies did not pertain to OHVIRA, and so subsequent search terms were specified. The study selection process using these search terms is shown in Figure 1. When narrowing the search to “endometriosis and Herlyn–Werner–Wunderlich syndrome”, only 24 studies were retrieved. Similarly, using the terms “endometriosis and OHVIRA syndrome”, 20 studies were identified, while the search term “uterus didelphys, renal agenesis and endometriosis” yielded 30 studies. Therefore, 74 publications were identified in the initial search in both databases using the three different search term combinations. A comparison of the search results from both databases revealed 28 duplicates (*n* = 10 studies on OHVIRA and *n* = 18 studies on OHVIRA and uterus didelphys, renal agenesis and endometriosis) that were excluded, and the full texts of the remaining 46 studies were screened for eligibility. In this step, a further ten studies were removed based on the inclusion and exclusion criteria (language: *n* = 3, video material: *n* = 2, review: *n* = 4, letter to the editor: *n* = 1). Therefore, 36 studies were included in the final analysis. The included studies were assessed for the following parameters: existing cases of uterine malformations associated with renal agenesis, age distribution at menarche, diagnostic methods (visual inspection, biopsy), patient symptomatology, distribution of malformations and types of surgical treatment administered. The studies were further examined to determine whether the authors performed a diagnostic laparoscopy, cystectomy, adhesiolysis, hysterectomy, vaginoplasty or surgical examination to diagnose and/or treat endometriosis. A focused investigation was specifically conducted on cases involving renal agenesis and uterine malformation to determine whether any abdominal interventions were performed and to explore any potential associations with endometriosis.

## 3. Results

The initial literature search with a broader search term combination (“endometriosis and urogenital malformation”) yielded many unspecific and irrelevant results, so we decided to narrow the search to a particular urogenital anomaly in the context of endometriosis, OHVIRA. The results presented here, therefore, pertain to adolescent patients with OHVIRA or a similar anomaly and endometriosis.

The findings of the 36 finally included studies are presented in Table 1. These studies entailed a total of *n* = 563 cases. Of the 36 studies, 21 reported a single case [31,32,33,34,35,36,37,38,39,40,41,42,43,44,45,46,47,48,49,50,51], 1 study reported two cases [52], 1 study reported four cases [53], 1 study reported five cases [54] and 12 studies presented ten or more cases [15,23,25,55,56,57,58,59,60,61,62,63]. The majority of patients were in their adolescence. The most prevalent symptoms included dysmenorrhea, vague lower abdominal pain and foul-smelling vaginal discharge after menstrual periods.

Details about abdominal exploration via laparoscopy or laparotomy were available for 97 cases, as abdominal intervention was unnecessary in most instances. Information regarding ipsilateral renal agenesis (IRA) was available for *n* = 275 cases. IRA occurred on the right side in *n* = 168 cases (61%), on the left side in *n* = 107 cases (39%), and no specific side was indicated for the remaining cases. Most cases exhibited congenital malformations in the development of the Wolffian and Müllerian ducts, characteristic of the triad consisting of obstructed hemivagina, ipsilateral renal anomaly and uterus didelphys [64]. Information about endometriosis involvement was found in 20 studies [23,25,31,32,33,36,43,44,46,47,52,53,54,55,56,57,58,59,60,61].

Effective treatment involved vaginal septoplasty for the symptom management of imperforate vagina. Among these 97 cases, endometriosis was identified in 61 (62.9%), compared to a rate of approximately 10% in women of reproductive age. Studies with larger case numbers, such as Acién P. et al., demonstrated a 20% endometriosis rate in 60 cases with bicornuate/didelphys uterus [55]; Tong J. et al. reported an endometriosis rate of 17.1% (*n* = 12) in *n* = 70 cases, with abdominal exploration mentioned in only 9 cases [60]. One year later, Tong et al. observed an endometriosis rate of 19.15% (18/94) in patients with OHVIRA, where patients with complete hemivaginal obstruction exhibited a higher rate of 37.0% (10/27) compared to those with incomplete obstructions at 11.9% (8/67) [23]. Another study on this malformation was conducted by Fedele et al. In their case series from 1981 to 2011, they examined 87 female patients and described the various anatomical variants of uterus didelphys with unilateral cervico-vaginal obstruction and ipsilateral renal anomalies and highlighted the significant clinical and surgical implications of these malformations. They reported a higher incidence of unilateral obstruction on the right side: right-sided in 53 of 87 cases (60.9%) and left-sided in 34 of 87 cases (39.1%). All patients underwent diagnostic laparoscopy to assess the uterine morphology and confirm the external uterine profile. Although the main aim of their study was not to investigate the association between HWWS and endometriosis, endometriosis was diagnosed in 12 of the 87 patients (13.8%), in 10 cases on the peritoneum, in 5 cases on the ovary and in 1 case each on the fallopian tube and diaphragm [65].

The studies assessed in the present review show a frequent association between genitourinary malformation and endometriosis [6,8]. In their case series on urogenital malformations, Moon et al. report the outcomes of five patients, averaging 16.6 years of age (range 13–27). Among the subset of four patients requiring laparoscopic intervention due to these malformations, three exhibited evidence of endometriosis [54].

In their study involving 60 patients with a mean age of 26.6 years (range 11–62), Acién et al. reported an endometriosis rate of 20% [55]. In another paper, Tong et al. documented an endometriosis rate of 17.1% in patients with a mean age of 21.39 years (range 10–50), with abdominal exploration mentioned in only 12.9% of cases [60]. One year later, Tong et al. observed that the diagnosis of endometriosis occurred at a mean age of 17.8 years, while OHVIRA was diagnosed at 20.5 years. They identified an endometriosis rate of 19.15% in patients with OHVIRA, noting a higher rate of 37.0% in those with complete hemivaginal obstruction, compared to those with incomplete obstruction (11.9%) [23]. It should be noted that, in these three studies, it is not clear whether all patients underwent abdominal exploration as a means of assessing for endometriosis [23,55,60]. The majority of the 563 patients included in this review found transvaginal septum resection and vaginoplasty to be a sufficient treatment, with only 97 cases requiring laparoscopic or laparotomic intervention. Among the 97 patients requiring abdominal exploration, endometriosis was diagnosed in 61 (62.9%).

## 4. Discussion

OHVIRA describes the association of uterus didelphys with ipsilateral vaginal obstruction and ipsilateral renal agenesis [22]. Renal agenesis is present in 30% of cases of uterine didelphys and is the most common anomaly [20]. Renal agenesis often occurs on the right side. Several studies suggest a prevalence of right renal agenesis in 66–75% of cases [22,66,67]. In our review, we also observed a higher prevalence of renal agenesis on the right side.

The results of our literature review revealed that rare urogenital malformations are increasingly being diagnosed prenatally or in early childhood, partly owing to advancements in ultrasound imaging quality and sensitivity, enhanced expertise in examinations and the increasing use of ultrasound in both prenatal diagnostics and routine clinical assessments [63]. Affected individuals typically remain asymptomatic prior to menarche [15,68]. In their review, Lecka-Ambroziak et al. also emphasize the necessity of screening for congenital anomalies of the genital tract in patients diagnosed with renal anomalies and vice versa, to recognize the consequences of these malformations and potentially avoid emergency surgeries [17]. Symptoms, such as progressive lower abdominal pain, dysmenorrhea, hematocolpos, pelvic mass and, in some cases, abnormal vaginal discharge, vomiting, fever, acute urinary retention or infection manifest only after menarche [15,69,70]. Surgical intervention is generally recommended after menarche, aiming to treat symptoms, alleviate obstructions, prevent complications and preserve fertility. The standard surgical procedure involves a transvaginal one-stage drainage of the hematocolpos/hematometrocolpos, vaginal septum resection and vaginoplasty (a restoration of the continuity of the vaginal mucosal wall to minimize the risk of postoperative stenosis and adhesions). Abdominal exploration in OHVIRA syndrome is reserved for select cases, such as for defining the anatomy more precisely, confirming the presence of endometriosis, treating a residual ureter or Gartner’s cyst or addressing symptoms resistant to therapy and findings requiring further clarification [15].

The cause of increased endometriosis risk in cases of urogenital malformations is still unclear, and while retrograde menstruation might certainly play a role, genetic, immunological and environmental factors likely contribute. These factors might also explain the occurrence of urogenital malformations. The concomitant occurrence of both entities could be explained by the fact that the anatomical and physiological changes associated with uterine malformations cause an impairment of uterine peristalsis, resulting in the occurrence of more frequent subendometrial myometrial contraction waves than in healthy individuals [71,72]. Due to the obstruction and/or stenosis of the cervical canal, this could cause increased resistance to the outflow of blood through the cervical canal and thus to increased retrograde menstruation, which, in turn, leads to the development of endometriosis [8,73,74]. Genetic predisposition is also a risk factor for endometriosis [75,76,77]. There is a possibility that genetic factors acting together in both conditions might provide an additional plausible explanation for the association between them.

One key problem in the diagnosis of endometriosis is the time delay between the first symptoms to histopathological confirmation. Sonography is the gold standard for evaluating the pelvic organs and is an excellent tool in the diagnosis of genitourinary malformation [20]. If vaginal sonography is refused or not feasible for any reason, transrectal sonography might be a feasible alternative, with very good diagnostic value. Renal sonography is an important standard examination to monitor the kidney or, in rare cases, to evaluate renal agenesis. If uterine didelphys and renal agenesis are present, an MRI of the pelvic abdomen should be performed for radiological assessment, differential diagnosis and the exclusion of other malformations. Renal scintigraphy to exclude a functional abnormality in the existing kidney and ureter is also required [20,25]. In therapy-resistant symptomatic adolescent patients with a clinically identified genitourinary malformation, a laparoscopic examination could be performed to detect the malformation of the uterus and to exclude endometriosis and pelvic adhesions that cannot be visualized via imaging [78]. Based on the majority of studies identified in this review, reporting single cases only, a clinical recommendation on whether or not to perform laparoscopy cannot be deduced.

The early diagnosis and appropriate treatment of endometriosis can prevent further destruction of the infiltrated organs and normal anatomy. Involvement of the ureter in endometriosis is a rare but serious condition that can lead to significant health problems. When the ureter is strangulated by endometriosis, hydronephrosis and ultimately renal failure might occur [79,80,81,82]. This is especially important in renal agenesis, as patients have only one kidney, and renal loss would be life-threatening. The appropriate symptomatology through guided clinical examination enables timely diagnosis and the appropriate, correct planning of further treatment. Laparoscopic repair of symptomatic endometriosis significantly improves quality of life and enables the avoidance of endometriosis-related complications and late sequelae.

### Limitations

As abdominal exploration was not required in all cases, and our analysis was limited to those cases where practitioners deemed abdominal exploration necessary and subsequently performed surgery, the reported prevalence might be overestimated. Despite this, our findings align with the existing literature, which still implies a significant association, indicating an increased risk of endometriosis in cases of urogenital malformations [6,23,25,55,56,57,60]. Another limitation of this study is the fact that our search study might have been biased for cases with concomitant endometriosis and urogenital malformations, potentially resulting in an overestimation of endometriosis prevalence in the targeted population. Lastly, the rarity of HWWS and endometriosis occurring concomitantly limits the amount of controlled studies with large patient volumes and, therefore, many findings, and the conclusions are based on case reports and case series.

## 5. Conclusions

Endometriosis appears to occur earlier in adolescents with concomitant HWWS, and this points to the need for a timely and specific evaluation of patients presenting with such anomalies. Adolescents with severe dysmenorrhea should be evaluated for urogenital malformation and endometriosis. Sonography provides a good diagnostic tool to plan further diagnostic and surgical treatment to avoid the late effects of DIE.

## Figures and Tables

**Table 1:** Studies on adolescent patients with urogenital malformations and endometriosis.

Study	Country	Patients	Symptoms	Age at Diagnosis in Years	Age at Menarche in Years, Menstrual Cycle	Renal Agenesis Right (R)/Left (L)	Malformation	Treatment	Abdominal Exploration No (N), Yes (Y), Not Available (NA)	Endometriosis
**Studies with ≥50 patients**										
Kapczuk K. et al., 2023 [57]	Poland	50	15/50 anomalies associated with cryptomenorrhea, and 35/50 were menstruating	13.5 (range 11.1–18.5)	2 girls detected in childhood	27 (15R/12L)	27/50 IRA; 22/27 UD, OHV; 1/27 UD with unilateral cervical atresia; 4/27 unicornuate uterus with RUH	17/50 only VSR (9/17 with IRA); 33/50 AE with LSC or laparotomy (18/33 with IRA)	33/50 Y	24/33 endometriosis (15/18 with IRA)
Acién P. et al., 2010 [55]	Spain	60	pelvic pain/dysmenorrhea, metrorrhagia	26.6 (range 11–62)	NA	60 (32R/28L)	60 IRA; 10 (8R/2L) UD; 8 (5R/3L) bicornis–unicollis uterus; 27 (15R/12L) bicornis–bicollis uterus; 10 (2R/8L) unicornuate uterus	HSG and/or LSC	NA	Bicornuate/didelphys uterus 20% Endometriosis
Tong J. et al., 2013 [60]	China	70	45/70 (64.3%) dysmenorrhea; 28/70 (40.0%) intermittent mucopurulent discharge; 18/70 (25.7%) metromenorrhagia	21.39 (range 10–50)	NA	70 (42R/28L)	70 IRA, UD, OHV (20/70 complete obstruction with HC; 50/70 incomplete obstruction)	all patients VSR; 9/70 (12.9%) underwent AE via laparotomy or laparoscopy	9/70 Y	Endometriosis was observed in 12 (17.1%) patients who underwent AE
Tong J. et al., 2014 [23]	China	94	NA	endometriosis 17.8 (range 13–32) HWWS 20.5 (range 13–37)	NA	NA	NA	NA		Pelvic endometriosis in 18/94 (19.15%) of patients with HWWS (patients with complete hemivaginal obstruction 10/27 (37.0%) higher than in those with incomplete obstructions 8/67 (11.9%))
Fei F.Y., 2022 [63]	USA	125	abdominal pain and/or dysmenorrhea	14.5 ± 6.5 (r 0–32)	12.3 ± 1.6 (r 9–19)	NA	VS 41.6% (52/125), including 39/52 with oblique septum or obstructed hemivagina–ipsilateral renal agenesis (OHVIRA)	45 VSR	N	NA
**Case series with ≤28 patients**										
Güdücü N. et al., 2012 [52]	Turkey	2	Pat I: dysmenorrhea, Pat. II: foul-smelling vaginal discharge after her menstrual periods	13, 21	Pat. I: 12; regular Pat. II: NA	2 R	Pat. I + II: UD, right OHV with HC, left uterine cavity, cervix and vagina normal	Pat. I: laparotomy, hemi-hysterectomy with salpingectomy. Par. II: VSR	Pat I:Y/Pat. II:N	Pat. I: DIE endometriosis, Pat. II: AE not performed
Kimble R. et al., 2009 [53]	Austalia	4	dysmenorrhea, abdominal pain	14 (range 12–17)	11,7 (range 10–14)	4 (3R/1L)	4 IRA, UD, OHV	LSC	Y	2 endometriosis, 2 no endometriosis
Moon L.M. et al., 2023 [54]	USA	5	dysmenorrhea (80%), irregular bleeding (40%), acute onset left lower quadrant pain (20%), and abdominal mass (20%)	16.6 (r 13–27)	12 (r 11–13)	5 (1R/4L)	5 IRA, UD, (3 left uterine horn distended with HM and HS, 3 left cervico-vaginal agenesis, 1 left cervical dysgenesis, 1 right cervical dysgenesis)	3/5 LSC (2, robotic) uterine horn resection, 1/5 Cervico-vaginal anastomosis + LSC ovarian cystectomy 1/5 cervico-vaginal anastomosis	4Y, 1N	3 endometriosis
Kudela G. et al., 2021 [58]	Poland	10	9/10 (90%) lower abdominal pain; 1/10 asymptomatic	13.35 (range 11–15)	mean 12.55	10 (7R/3L)	10 IRA, UD, OHV	1/10 LSC hemihysterectomy and vaginectomy; 1/10 LSC conversion to laparotomy hemihysterectomy, vaginectomy and salpingectomy; 8/10 only VSR	2/10 Y	1 endometriosis, 1 no endometriosis
Sabdia S. et al., 2014 [59]	Austalia	10	dysmenorrhea	12 (range 10–14)	NA	10 (7R/3L)	NA	8 VSR, 1 vs. excision, 2 patients with cervical aplasia geht laparoscopic hemi-hysterectomy	NA	6 endometriosis
Stassart J.P. et al., 1992 [25]	USA	15	dysmenorrhea and increasing pelvic pain	14.5 (range 6–26)	11,7 (range 10–15), 1 premenarch	15 (11R/4L)	15 IRA, UD, OHV (9/15 complete obstruction with HC; 6/15 incomplete obstruction)	13/15 underwent AE, 2/15 not AE	13/15 Y	7/13 endometriosis
Zhang H. et al., 2017 [61]	China	21	16/21 primary amenorrhoea with cyclic pelvic pain; 5/21 progressive dysmenorrhea	14 (range 10–18)	NA	6 (4R/2L)	6/21 IRA; 4/6 UD; 2/6 unicornuate uterus	1 hymen incision, 4 vaginoplasty; 3 VSR, 11 resection of uterus, 1 resection of RUH, 1 hemihysterectomy	11/21 Y	6/11 endometriosis. 3/6 HWWS cases underwent AE and 2/3 were diagnosed with endometriosis
Kapczuk K. et al., 2017 [56]	Poland	22	dysmenorrhea	13.1 (range 11.4–18.2)	12.2 (range 11–15.6)	22 (13R/9L)	22 IRA; 18/22 (81.8%) UD + OHV; 1/22 (4.5%) UD + unilateral cervical atresia; 3/22 (13.6%) unicornuate uterus + RUH with functional noncommunicating cavity	2/22 laparotomy + VSR; 2/22 LSC + VSR; 3/22 laparotomy + RUH removal, 1/22 cervical fenestration + hemihysterectomy; 13/22 only VSR	8/22 Y	4/8 pelvic endometiosis stage 1
Zhang J. et al., 2020 [62]	China	26	21/26 (80.8%) dysmenorrhea; 16/26 (61.5%) cystic mass; 11/26 (42.3%) irregular vaginal discharge; 6/26 (23.1%) prolonged menstrual periods	15.5 (range 10–31)	11.7 (range 10–15)	24 NA	24/26 IRA; 20/26: UD, 2/26 uterus bicornuate, 4/26 uterus septate. 25/26 OHV (17/25 complete obstruction; 8/25 incomplete obstruction)	25/26 VSR, 1/26 LSC hemi-hysterektomy	NA	NA
Zarfati A. et al., 2022 [15]	Italy	28	abdominal pain, dysmenorrhea, vaginal discharge, irregular menstruation, infection, palpable abdominal mass, rectal tenesmus	11.9 (r 0,5–15,7) (7/28 (25%) before menarche, 1/28 (3%) perinatally)	NA	23 (18R/5L)	23 IRA, 5 multicystic-dysplastic kidney	25/28 VSR, 1/28 VSR + LSC ovarian cystectomy (before menarche), 2/28 not yet operated on as not yet in menarche	1Y (before menarche), 27 N	No endometriosis
**Case reports with individual cases (*n* = 1)**										
Ahmad Z. et al., 2013 [31]	India	1	primary infertility	22	13; regular	L	IRA, UD, left OHV	laparotomy, cystectomy and adhesiolysis	Y	Endometrioma and peritoneal endometriosis
Altchek A. et al., 2009 [32]	USA	1	dysmenorrhea and right lower quadrant pain and rectal pressure	13	11; regular	R	IRA, UD, right HC + HM	LSC	Y	Peritoneal endometriosis
Arakaki R. et al., 2023 [33]	Japan	1	dysmenorrhea and abnormal vaginal discharge	12	11; regular	L	IRA, UD, left OHV	LSC	Y	Endometriosis at the left fallopian tube
Basnet T. et al., 2020 [34]	Nepal	1	mass associated with pain at vaginal introitus	17	14; irregularly	R	IRA, UD, right HC	VSR	N	AE not performed
Borges A.L. et al., 2023 [35]	Portugal	1	foul discharge, dysmenorrhea	17	11, regularly	L	IRA, UD, left OHV	VSR	N	AE not performed
Cheung V. et al., 2008 [36]	USA	1	dysmenorrhea, irregular menstrual cycles	17	NA	L	IRA, UD, cyst in the left ovary	LSC cystectomie	Y	Endometioma
Cox D. et al., 2012 [37]	USA	1	cyclic abdominal pain, enlarged abdominal mass	17	12; regular	L	IRA, UD, left OHV	VSR	N	AE not performed
Girardi Fachin C. et al., 2019 [38]	Brazil	1	dysmenorrhea, nausea, vomiting, fever and a palpable abdominal mass in the hypogastric region	12	11; irregular	L	IRA, uterus bicornis (left hemiuterus was distended and connected to the right one only by a myometrial strand)	LSC hemi-hysterectomy	Y	NA
Hayat A.M. et al., 2022 [39]	Afghanistan	1	dysmenorrhea	15	13; regular	1R	IRA, UD, right HC + HM, left uterine horn and cervical canal normal	VSR	N	NA
Khaladkar S.M. et al., 2016 [40]	India	1	dysmenorrhea	13	12; regular	1L	IRA, UD, left OHV, right uterine cavity, cervix and vagina normal	laparotomy, hemi-hysterectomy	Y	NA
Kulkarni A. et al., 2023 [41]	India	1	pelvic pain	12	premenarchal	1R	IRA, UD, right HC + HM; left uterine horn and cervical canal normal	vaginoscopic VSR	N	AE not performed
Mabuchi Y. et al., 2021 [42]	Japan	1	fever and lower abdominal pain	25	11; regular	1R	IRA, UD, right HC + HM; left uterine horn and cervical canal normal	vaginoscopic VSR	N	AE not performed
Miyazaki Y. et al., 2020 [43]	Japan	1	dysmenorrhea, cystic mass in the lower pelvic cavity	20	13; regular	1L	IRA, unicornuate uterus + RUH with functional noncommunicating cavity	LSC left endometrioma and hematosalpinx.	Y	Endometrioma
Nawfal K. et al., 2011 [44]	USA	1	dysmenorrhea	20	12; irregular	1R	IRA, UD, OHV, HC	LSC, supracervical hemi-hysterektomy right with in-situ pregnancy	Y	Peritoneal endometriosis
Nishu D.S. et al., 2019 [45]	Bangladesh	1	dysmenorrhea, right lower abdominal pain	15	15; regular	1R	IRA, UD, OHV, HC	vaginoplasty VSR	N	AE not performed
Patterson D. et al., 2006 [46]	USA	1	progressive cyclic rectal pain, chronic constipation	10	NA	1R	IRA, UD, OHV, HC+ HM	LSC and vaginoscopy	Y	No endometriosis
Shah D.K. et al., 2011 [47]	USA	1	dysmenorrhea and metrorrhagia	12	NA	1R	IRA, UD, OHV, HM	VSR, 2 years later LSK	Y	Endometriosis stage 1
Shim J. et al., 2020 [48]	USA	1	dysmenorrhea and increasing pelvic pain	15	14	1R	IRA, UD, HM, OHV with communication between the right hemivagina and the left cervix	robot-assisted hemihysterectomy and fallopian tube	Y	NA
Sijmons A. et al., 2023 [49]	Netherlands	1	anuria and intralabial mass	1 day		1R	multicystic dysplastic kidney, UD, OHV and an ectopic ureteric insertion.	hymen incision, nephrectomy	NA	NA
Widyakusuma L.S. et al., 2018 [50]	Indonesia	1	acute pelvic pain, dysmenorrea, cystic mass with a smooth surface in the right lateral region of the midline	23	12; regular	1R	1 IRA, UD, OHV	VSR	N	NA
Yamada Y. et al., 2023 [51]	Japan	1	dysmenorrhea and lower abdominal pain	12	12	1R	1 IRA, UD, OHV	vaginoscopic VSR	N	NA

IRA: ipsilateral renal agenesis, UD: uterus didelphys, RUH: rudimentary uterine horn, HC: hematocolpos, HM: hematometra; HS: hematosalpinx, OHV: obstructed hemivagina, VS: vaginal septum, AE: abdominal exploration, VSR: vaginal septum resection, HSG: hysterosalpingography, LSC: laparoscopy, NA: not available.

## Data Availability

Publicly available datasets were analyzed in this study.
